# The soybean GmDi19-5 interacts with GmLEA3.1 and increases sensitivity of transgenic plants to abiotic stresses

**DOI:** 10.3389/fpls.2015.00179

**Published:** 2015-03-24

**Authors:** Zhi-Juan Feng, Xiao-Yu Cui, Xi-Yan Cui, Ming Chen, Guang-Xiao Yang, You-Zhi Ma, Guang-Yuan He, Zhao-Shi Xu

**Affiliations:** ^1^The Genetic Engineering International Cooperation Base of Chinese Ministry of Science and Technology, Key Laboratory of Molecular Biophysics of Chinese Ministry of Education, College of Life Science and Technology, Huazhong University of Science and TechnologyWuhan, China; ^2^Institute of Crop Science, Chinese Academy of Agricultural Sciences/National Key Facility for Crop Gene Resources and Genetic Improvement, Key Laboratory of Biology and Genetic Improvement of Triticeae Crops, Ministry of AgricultureBeijing, China; ^3^College of Life Sciences, Jilin Agricultural UniversityChangchun, China

**Keywords:** Di19 protein, genome-wide analysis, stress response, functional identification, protein interaction, *Glycine max*

## Abstract

Drought-induced (Di19) proteins played important roles in plant growth, development, and abiotic stress responses. In the present study, a total of seven *Di19* genes were identified in soybean. Each soybean *Di19* gene showed specific responses to salt, drought, oxidative, and ABA stresses based on expression profiles. With a relatively higher transcript level among *Di19* members under four stress treatments, *GmDi19-5* was selected for detailed analysis. Inhibitor assays revealed that ABA inhibitor (Fluridone) or H_2_O_2_ inhibitor (DMTU) was involved in the drought- or salt-induced transcription of *GmDi19-5*. The GUS activity driven by the *GmDi19-5* promoter was induced by salt, PEG, ABA, and MV treatments and tended to be accumulated in the vascular bundles and young leaves. A subcellular localization assay showed that GmDi19-5 protein localized in the nucleus. Further investigation showed that GmDi19-5 protein was involved in the interaction with GmLEA3.1. Overexpression of *GmDi19-5* increased sensitivity of transgenic *Arabidopsis* plants to salt, drought, oxidative, and ABA stresses and regulated expression of several ABA/stress-associated genes. This present investigation showed that *GmDi19-5* functioned as a negative factor under abiotic stresses and was involved in ABA and SOS signaling pathway by altering transcription of stress-associated genes.

## Introduction

Abiotic stress may occur at any stage of plant development and often several types of stresses occur simultaneously (Rizhsky et al., 2004). To reduce the adverse effects of stress, plants have evolved multifaceted strategies, including morphological, physiological, and biochemical adaptations (Ingram and Bartels, 1996; Xiong et al., 2002; Zhu, 2002; Shinozaki et al., 2003; Bohnert et al., 2006). It is also demonstrated that plants have evolved a complex and elaborate signaling network that perceives and responds to continuously changing surroundings by modulating the expression of downstream genes (Xu et al., 2011). It has been demonstrated that the modulation of signaling regulators will be a promising method for improving the stress tolerance of plants. A number of stress-regulated genes encode regulatory proteins, such as transcription factors, that are important in regulating the expression of downstream genes (Seki et al., 2002; Singh et al., 2002; Lee and Lee, 2003; Rabbani et al., 2003). Zinc finger proteins are one of the major families of eukaryotic transcription factors (Klug and Schwabe, 1995; Takatsuji, 1999; Englbrecht et al., 2004). Within this family, the Cys2/His2-type (C2H2) zinc finger proteins contain one or more tandem of C2H2 zinc finger motifs, one of the best-characterized DNA-binding motifs. This motif contains two cysteines and two histidines that serve as zinc ligands and is represented by the signature Cys-X_2,4_-Cys-X_12_-His-X_3,4,5_-His (Brown et al., 1985; Miller et al., 1985; Pabo et al., 2001; Sakamoto et al., 2004). Drought-induced (Di19) proteins are zinc finger transcription factors that play important roles in development, growth, and response to stress (Milla et al., 2006; Li et al., 2010a,b; Liu et al., 2013b; Qin et al., 2014). Di19 proteins are encoded by a small gene family that numbers seven isoforms in *Arabidopsis* and five in rice (Milla et al., 2006). Di19 proteins are known also in cotton (Li et al., 2010a) and wheat (Li et al., 2010b).

The Di19 family functionally participated in various signaling pathways. Di19s have been found acting as both transcription repressors and activators (Li et al., 2010a,b; Liu et al., 2013b; Qin et al., 2014). In *Arabidopsis, AtDi19-1* and *AtDi19*-3 were rapidly induced by drought stress, whereas transcripts of *AtDi19-2* and *AtDi19-4* were accumulated at high levels by high salinity stress (Milla et al., 2006). *AtDi19-7* has been implicated in regulating light signaling, and did not respond to abiotic stress treatments (Kang et al., 2005). These findings indicate that in the *Di19* family, different members may respond to different signal stimuli and accomplish specific functions.

Cys2/His2-type zinc finger proteins can bind to DNA elements (Searles et al., 2000; Wolfe et al., 2000; Liu et al., 2013b; Qin et al., 2014). In *Arabidopsis*, both AtDi19-1 and AtDi19-3 could bind to the TACA(A/G)T element (Liu et al., 2013b; Qin et al., 2014). Further assays demonstrated AtDi19-1 could directly up-regulate the expressions of *PR1, PR2*, and *PR5* in response to drought stress. In addition to binding DNA elements (Liu et al., 2013b), Cys2/His2-type zinc finger proteins may also participate in protein-protein interactions (Fukamatsu et al., 2005; Milla et al., 2006; Liu et al., 2013b). In *Arabidopsis*, AtDi19-1 protein interacted with CPK11 (a calcium-binding protein kinase) and its transactivation activity could be enhanced through phosphorylation by CPK11 at the nuclear location signal (NLS)-containing motif (Milla et al., 2006; Liu et al., 2013b). This suggested that posttranslational modification might be important to regulate the function of Di19 protein (Milla et al., 2006). AtDi19-7 interacted with F-box AtLKP2 protein that might be light receptors and function within or very close to the circadian oscillator (Kiyosue and Wada, 2000; Somers et al., 2000; Imaizumi et al., 2003; Fukamatsu et al., 2005). Protein-protein interactions are critically important to many processes that take place in the cell, such as signal transduction, and regulation of gene expression. Therefore, it is significant to identify interacting proteins of Di19s.

In this study, we characterized the Di19 protein family of soybean, by identifying its seven members and their chromosome locations, gene structures, and expression profiles. Moreover, *Di19-5* was selected for detailed functional analysis.

## Materials and methods

### Search and identification of Di19 family members

*Arabidopsis* Di19 (Milla et al., 2006) sequences were retrieved from the *Arabidopsis* Information Resource (http://www.arabidopsis.org) and used to search homologous Di19s from the soybean database (http://www.phytozome.org/) (Release 9.1). BLASTN and BLASTP programs were used to identify homologous EST singletons and peptides, respectively. Redundant sequences were removed via the decrease redundancy tool (http://web.expasy.org/decrease_redundancy/). Each non-redundant sequence was checked for the presence of two conserved C2H2 zinc finger domains.

### Phylogenetic tree and sequence alignments

The phylogenetic tree of Di19s was constructed using the neighbor-joining method in Molecular Evolutionary Genetics Analysis (MEGA; version 4.1) with the following parameters: Test Neighbor-Joining model and 1000 bootstrap replicates. Multiple sequence alignments were performed using the amino acid sequences of the conserved region and full-length protein by ClustalX2.0 software. Multiple Expectation maximization for Motif Elicitation (MEME) was used to identify the motifs of candidate Di19 proteins. Potential nuclear localization sequences (NLS) and putative nuclear export signal sequences (NES) were predicted by PSORT and NetNES software, respectively (Nakai and Kanehisa, 1992). The subcellular localization was predicted at YLoc (http://abi.inf.uni-tuebingen.de/Services/YLoc/webloc.cgi). Phosphorylation sites were predicted at NetPhos 2.0 Server (http://www.cbs.dtu.dk/services/NetPhos/).

### Chromosomal distribution, gene structure, and promoter region prediction

Chromosomal distribution was determined by searching the database containing the complete genome sequence of each soybean chromosome (http://www.phytozome.org/). Exon/intron gene structures were constructed by comparing the CDSs with their corresponding genomic DNA sequences and analyzed using the Gene Structure Display Serve tool (http://gsds.cbi.pku.edu.cn/).

To analyze their promoter regions, the 1.8 kb upstream regions of the genes, according to the position of the genes provided by the soybean annotation information, were selected and screened against the PLACE database (Higo et al., 1999).

### Soybean stress treatments

Soybean cultivar “Tie feng 8,” with characteristic of salt tolerance, was used in this study. Soybean seeds were grown in pots of peat/vermiculite (1:1 v/v) under conditions of 12 h of light followed 12 h of dark, constant temperature 25°C, and humidity 70%. Salt, drought, H_2_O_2_, and abscisic acid (ABA) stresses were applied to 2-week-old soybean seedlings. For salt stress, the roots of seedlings were dipped into solutions of 200 mM NaCl. For dehydration, the root systems of whole plants were washed gently with water to remove soil, and then the plants were put on filter paper for induction of a rapid drought treatment. For H_2_O_2_ stress, the roots of seedlings were dipped into solutions of 25 mM H_2_O_2_. For ABA treatment, soybean seedlings were sprayed with 100 μM ABA. For inhibitors assay, the plants were pretreated with H_2_O_2_ scavenger [10 mM dimethyl thiourea (DMTU)] and ABA scavenger (100 μM fluridone) for 6 h, respectively, and then exposed to dehydration and salt treatments for 0.5, 5, or 12 h. In order to get reliable results for all of the above treatments, the un-treated soybean seedlings with consistent growth were used as control for each series of treatments. At various time points after each treatment soybean seedlings were harvested, frozen in liquid nitrogen, and stored at −80°C until extraction of total RNA for qRT-PCR assays.

### Subcellular localization

*GmDi19-5* was inserted into the subcellular localization vector p16318 containing the CaMV 35S promoter and *green fluorescent protein* (*GFP*) gene. To obtain Di19 coding sequence without the stop codon, *Di19-5* cDNA was ligated into the *Hind* III/*Xba* I sites of p16318GFP vector (for primer sequences, Supplementary Table S1), upstream to the N-terminal end of GFP under control of the 35S promoter. Subcellular localization of transiently expressed GmDi19-GFP was assessed after transformation by particle bombardment into onion epidermal cells (Xu et al., 2007; Li et al., 2013). Fluorescence was observed by confocal laser scanning microscopy (LSM700; Carl Zeiss) after incubation at 25°C for 24 h on MS medium under dark conditions.

### Generation and stress treatments of transgenic arabidopsis plants

To generate *Arabidopsis* transgenic plants constitutively overexpressing the *GmDi19-5* gene, the coding sequence of *GmDi19-5* was cloned into the pBI121 vector with *Sma* I/*Sac* I sites to replace the *GUS* gene under the control of the CaMV 35S promoter (for primer sequences, Supplementary Table S1). To develop *Arabidopsis* transgenic plants expressing the GUS reporter gene under the control of the *GmDi19-5* promoter, the promoter sequence of *GmDi19-5* was cloned into the pBI121 vector with *Hind* III /*Xba* I sites to replace CaMV 35S promoter and fused to the N-terminal end of GUS (for primer sequences, Supplementary Table S1). Each construct was then transferred into *Arabidopsis* by the floral dip method. *Arabidopsis* lines carrying promoter CaMV 35S-GmDi19-5 gene were used for phenotypic analysis (Zhang et al., 2004). *Arabidopsis* lines (proDi19-5) carrying promoter Di19-5-GUS gene were used for expression analysis.

Seeds of transgenic overexpressing *Arabidopsis* and WT plants were grown on 10 × 10 cm MS agar plates. They were routinely kept for 3 d in darkness at 4°C to break dormancy and transferred in a tissue culture room under a day/night 16/8 h cycle at 22°C. For seed germination, a total of 50 seeds of transgenic lines or WT were kept on MS media supplemented with 50 mM NaCl, 2% PEG, 1.0 μM methylviologen (MV), or 1.5 μM ABA for 5 d. Seeds were considered germinated when radicles had emerged from the seed coat. For root growth, 30 5-d-old seedlings with consistent growth state of transgenic lines or WT were transferred to MS agar plates containing 100 mM NaCl, 4% PEG, 5 μM MV, or 10 μM ABA for 5 d. Seed germination rates and root lengths were analyzed. Each treatment contained three independent replicates.

### Screening of cDNA libraries and yeast two-hybrid interaction assay

The soybean seedling cDNA library was constructed in a pGADT7-Rec2 vector containing a GAL4 activation domain using Matchmaker Library Construction (Clontech) and then transformed into the yeast strain AH109 (Clontech). *GmDi19-5* was cloned into pGBKT7 bait vector (for primer sequences, Supplementary Table S1) and transformed into yeast strain Y187 (Clontech). Yeast two-hybrid screening was performed using the MATCHMAKER two-hybrid system (Clontech) as previously described (Liu et al., 2013a).

Candidates were retransformed with the bait vector into the yeast strain AH109 for two-hybrid analysis. Transformants were selected by growing on SD-Trp-Leu- at 30°C for 4 d. Surviving clones were retransferred to SD-Trp-Leu-His-Ade- medium according to the manufacturer's instructions (Clontech).

### Bimolecular fluorescence complementation (BiFC) assay

For BiFC analysis, the full-length coding sequence of *GmDi19-5* was cloned into PUC-pSPYNE vector and fused with the N-terminal fragment of YFP to form YFP^N^-Di19-5 construct (for primer sequences, Supplementary Table S1). The full-length coding sequence of *GmLEA3.1* was cloned into PUC-pSPYCE vector as a fusion with the C-terminal fragment of YFP to form YFP^C^-LEA3 construct (for primer sequences, Supplementary Table S1). For transient expression, plasmids of YFP^N^-Di19-5 and YFP^C^-LEA3 were co-transformed into onion epidermal cells by a particle gun and monitored by confocal microscopy as previously described (Liu et al., 2013a).

### Detection of β-glucuronidase (GUS) activity assay

For GUS activity assay, proDi19 transgenic seeds were germinated and grown on MS media for 5 d, and then exposed to MS media containing 100 mM NaCl, 4% PEG, 5 μM MV, or 10 μM ABA for 5 d to a tissue culture room under a day/night 16/8 h cycle at 22°C. Seedlings without any stress treatment were used as controls. Histochemical localization of GUS activities were analyzed after incubating the transgenic plants in 10 ml tubes with 1 mg/mL 5-bromo-4-chloro-3-indolyl-glucuronic acid, 5 mM potassium ferricyanide, 5 mM potassium ferrocyanide, 0.03% Triton X-100 and 0.1 M sodium phosphate buffer, pH 7.0 overnight at 37°C. Seedlings were cleared with 70% ethanol to remove chlorophyll from green tissue. GUS-stained plants were examined with a light microscope (Leica) at a low magnification and photographed with a digital camera.

### RNA isolation and quantitative real-time PCR (qRT-PCR)

Total RNA template was extracted from isolated different plant materials using Trizol reagent (Takara, Dalian, China) and reverse transcription was performed with 2 μg of total RNA for the first strand cDNA synthesis with a PrimeScript 1st Strand cDNA Synthesis kit (Takara, Dalian, China), according to the manufacturer's instructions. The qRT-PCR primers were designed from non-conserved regions of the genes (Supplementary Table S1). Soybean *Actin* (U60506) or *Arabidopsis Tublin* was used as internal controls for normalization of the template cDNA. qRT-PCR analyses were performed on an ABI7300 system with SYBR Premix ExTaq II (Takara, Dalian, China). The amount of transcript accumulated for GmDi19 genes or *Arabidopsis* stress-related genes normalized to the internal control gene was analyzed using 2^−ΔΔC_T_^ method (Livak and Schmittgen, 2001). Three independent experiments were accomplished and for each sample three technical replicates were analyzed.

### Statistical analysis

Statistical analyses were performed using the software in Excel. Analysis of variance was used to compare the statistical difference based on Student's *t*-test, at a significant level of *P* < 0.05, *P* < 0.01.

## Results

### Identification of the soybean Di19 family

Seven Di19s were identified in the soybean genome. They contained conserved two conserved C2H2 zinc finger domains which were consistent with *Arabidopsis* Di19 homologs (Supplementary Dataset S1). Based on suggested *Arabidops*is Di19 nomenclature, each gene was named with a two-letter code corresponding to *G. max* (Gm), followed the family designation (Di19), and finally a number (Table 1). The soybean Di19s encode proteins with predicted molecular mass of ~25 kD and isoelectric point (p*I*) < 7.

**Table 1:** **Nomenclature for Di19s in soybean**.

***GmDi19* gene name**	***GmDi19* gene model**	**Amino acid**	**Isoelectronic point (p*I*)**	**Molecular mass (kD)**	**Chromosome**
*GmDi19-1*	Glyma03g37530	218	4.66	24.35	3
*GmDi19-2*	Glyma19g40150	216	4.83	24.26	19
*GmDi19-3*	Glyma10g29030	219	5.52	24.35	10
*GmDi19-4*	Glyma15g15560	215	5.51	24.04	15
*GmDi19-5*	Glyma09g04490	215	5.38	24.07	9
*GmDi19-6*	Glyma07g32120	233	5.98	26.13	7
*GmDi19-7*	Glyma13g24420	237	6.12	26.40	13

### Phylogenetic tree and domain analysis of the Di19 family

**Figure 1:**
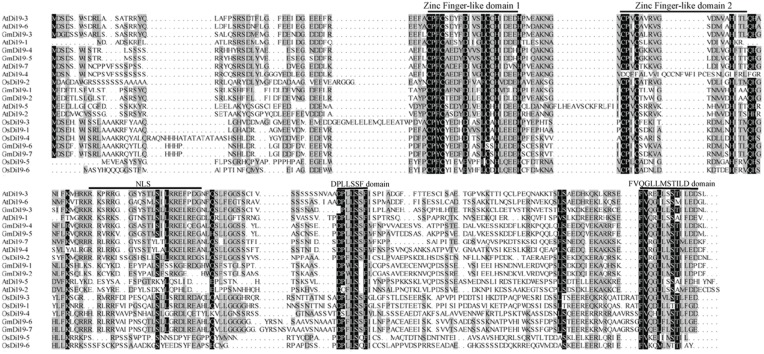
**Multiple alignments of soybean Di19s, AtDi19s, and OsDi19s**.

Multiple alignments were performed using the ClustalX program to examine sequence features of Di19 proteins. All soybean Di19 proteins contain highly conserved region with two unusual C2H2 zinc finger-like domains at the N-terminus, which span approximate about 60 amino acids (Figure 1). The two motifs differ slightly from the canonical C2H2 domain sequence (Cys–X_2−5_–Cys–X_12_–His–X_2−5_–His) (Klug and Schwabe, 1995). In soybean Di19 proteins, the spacing between the two Cys and two His amino acids in the first and second finger domain was 11 and 10 amino acids, respectively, as in *Arabidopsis* and rice Di19 proteins (Figure 1; Supplementary Dataset S2). Outside the putative zinc finger-like domain, a Leu-rich motif and two short regions in the C-terminus half of the proteins (consensus sequences “DPLLSSF” and “FVQGLLMSTILD”) were conserved among all members of the family (Figure 1). Members of the soybean Di19 family (except GmDi19-1 and GmDi19-2) contained NLS (Supplementary Table S2). Potential NES was also present in several soybean Di19 proteins (Supplementary Table S2). Subcellular localization predictions revealed that all soybean Di19 proteins were located in the nucleus (Supplementary Table S2). Moreover, a BLAST search against the NCBI translated database resulted in identification of homology proteins with the same features in *Arabidopsis*, rice, maize, and *Brachypodium* (Supplementary Table S3). MEME analysis identified 10 motifs (Supplementary Figure S1), of which motifs 1 and 2 were located in the conserved Di19 domain region of all Di19 proteins (namely the two C2H2 zinc finger-like domains). Domain analysis indicated that Di19 proteins were present and well conserved in both dicotyledonous and monocotyledonous plants (Supplementary Figures S1, S2; Supplementary Table S4). Different logos of these 10 motifs were shown in Supplementary Figure S2.

**Figure 2:**
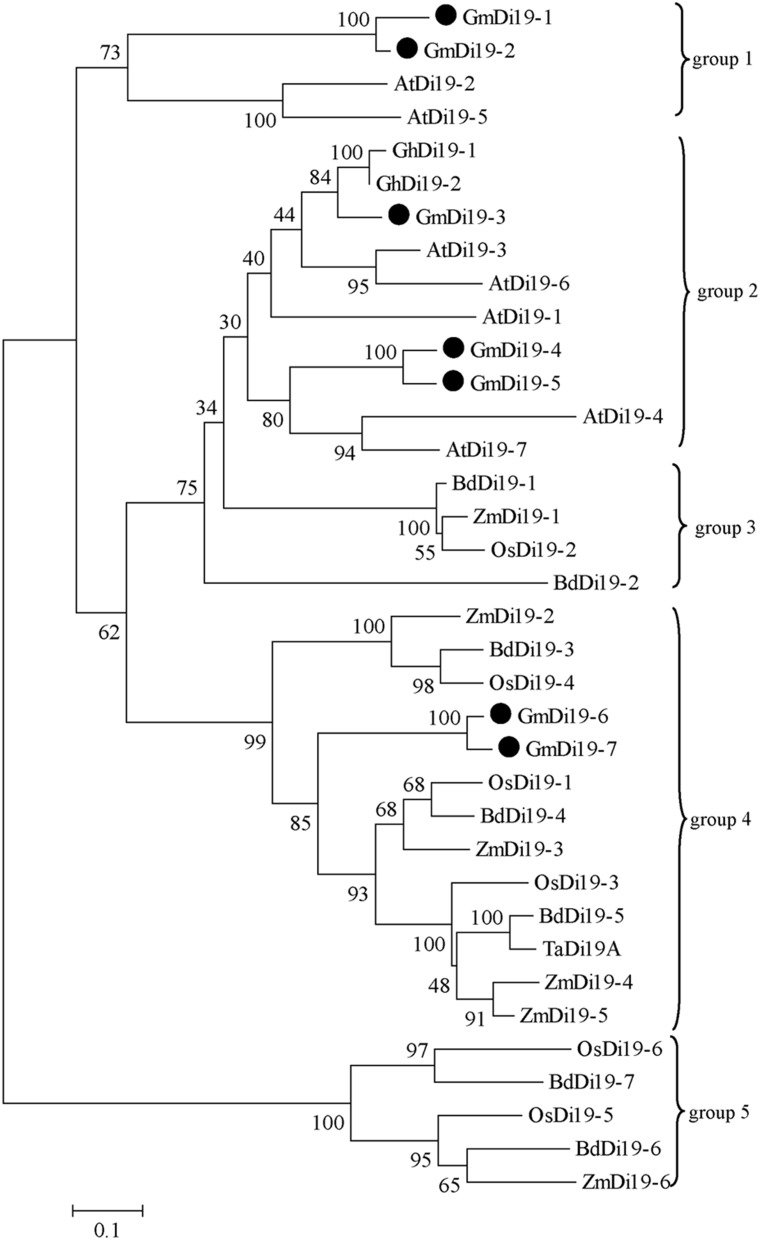
**Phylogenetic relationship of the soybean Di19s with those of *Arabidopsis*, rice, maize, *Brachypodium*, cotton, and wheat**.

Due to the high similarity of Di19 proteins, a phylogenetic tree was built on the basis of the full amino acid sequence of soybean, *Arabidopsis*, rice, maize, *Brachypodium*, cotton, and wheat Di19 proteins. Three pairs of closely related proteins were found: (i) GmDi19-1 and GmDi19-2, (ii) GmDi19-4 and GmDi19-5, and (iii) GmDi19-6 and GmDi19-7 (Figure 2). Phylogenetic tree showed all Di19s were divided into five groups (Figure 2). Dicotyledonous Di19s formed two groups 1 and 2. Monophyletic clade formed three groups 3, 4, and 5. The best orthology matches of the GmDi19-1, GmDi19-2, GmDi19-4, and GmDi19-5 proteins were AtDi19-2, AtDi19-5, AtDi19-4, and AtDi19-7, respectively. The best orthology matches of the GmDi19-3 protein were AtDi19-1, AtDi19-3, AtDi19-6, GhDi19-1, and GhDi19-2. The best orthology matches of the GmDi19-6 and GmDi19-7 proteins were ZmDi19-3, OsDi19-1, and BdDi19-4.

Additionally, the reliability of the phylogeny was further evidenced by parameters like motif compositions of individual subfamilies. Motif sequence conservation or variation between the proteins might specify the functional equivalence or diversification, respectively, with respect to various aspects of biological function. Members of a particular subfamily showed a tendency to have similar motif compositions and certain motifs were deleted or duplicated within particular clades. For example, motifs 7, 9, and 10 were absent from almost all members of Di19-group 1, and instead, Di19-group 2 included motif 9. Motif 7 only existed in Di19-group 4. Motifs 4, 5, 7, 8, and 10 were absent from almost all members of Di19-group 5 (Figure 2; Supplementary Figure S1). Specific motif deletion or duplication within a protein might be crucial for dispensing undesirable regions and maintaining or developing only those regions needed to develop a particular phenotype.

### Chromosomal location and gene structure analysis

The seven genes were located on different soybean chromosomes (Supplementary Table S3). However, two *Di19* genes were found on each of chromosomes 3 and 5 in *Arabidopsis*, three *Di19* genes were present on chromosome 5 in rice, and five were located on chromosome 2 in maize (Supplementary Table S3). Clearly, certain chromosomes in some species had a relatively high density of *Di19* genes (Supplementary Table S3).

**Figure 3:**
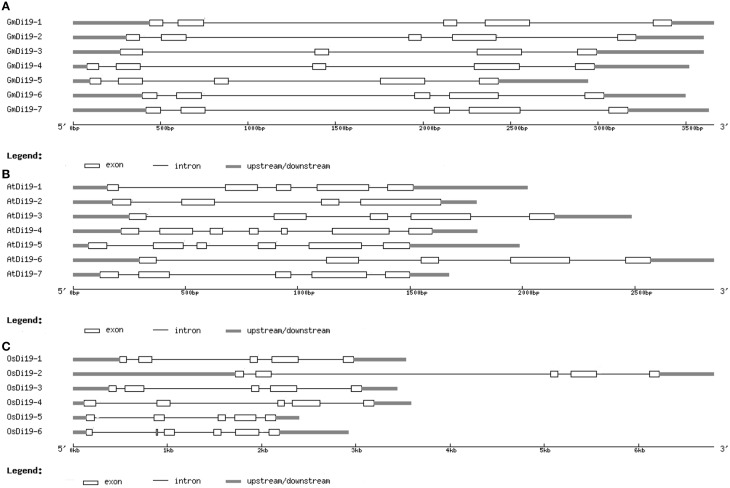
**Gene structures of soybean *Di19s* (A), *AtDi19s* (B), and *OsDi19s* (C)**. Exons and introns are represented by white boxes and blank lines, respectively.

To obtain some insight into the gene structures of the soybean *Di19* family genes, their exon/intron organizations were analyzed. All soybean *Di19* genes were disrupted by four or five introns (Figure 3A). Interestingly, each of the putative zinc finger domains in soybean Di19 proteins was encoded by the same adjacent exons (second and third exons). We also compared the gene structures of *Di19* genes in *Arabidopsis* and rice. The exon-intron structures were highly conserved in all cases (Figures 3B,C). These findings suggested that each subgroup of *Di19* genes was conserved in a relatively constant exon-intron composition during evolution.

### Abiotic stress responses

**Figure 4:**
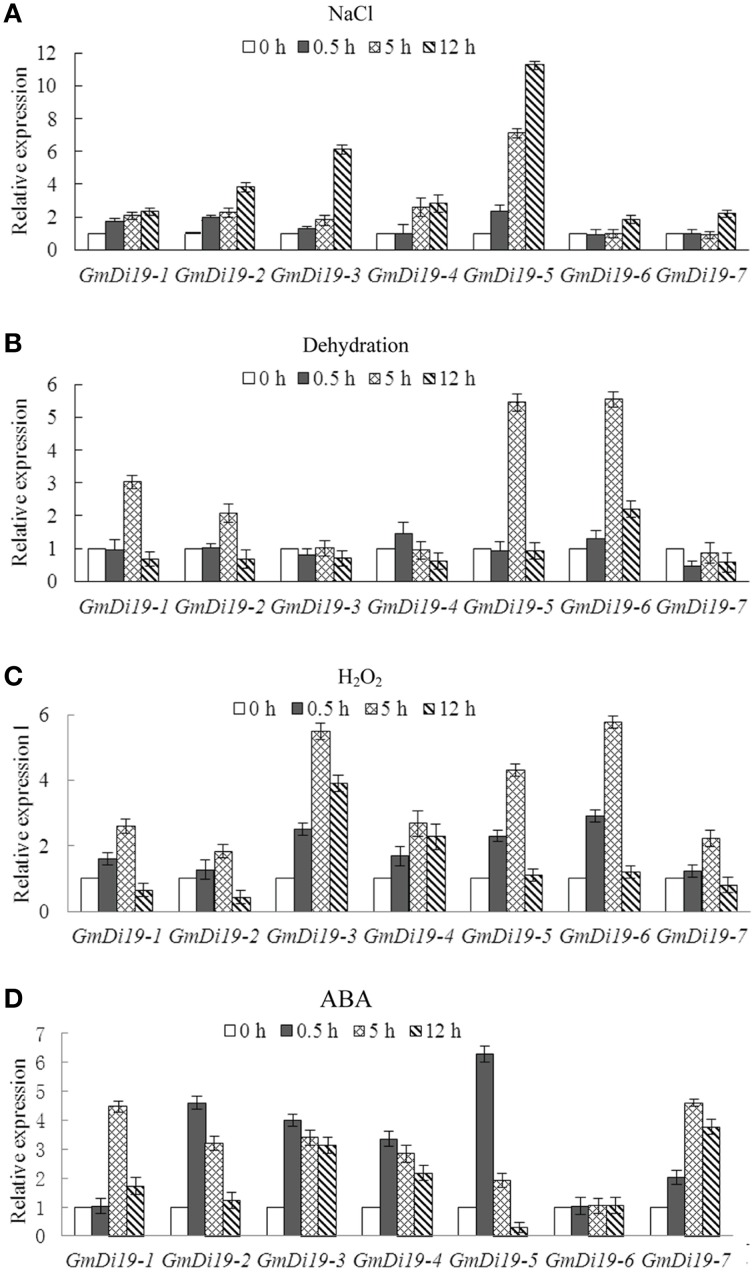
**Relative expression profiles of the *Di19* genes in the 2-week-old soybean seedlings subjected to NaCl (A), dehydration (B), H_2_O_2_ (C), and ABA (D) stress treatments**. The transcript levels of each Di19 in the stress-treated plants were plotted as the relative expression (fold) of the non-stressed control plants for 0.5, 5, and 12 h. The transcript level of *Actin* was used as a reference. Mean values and standard errors (bar) were shown from three independent experiments.

To investigate the effect of abiotic stresses on *Di19s*, expression pattern was measured by qRT-PCR in soybean seedlings subjected to salt, drought, H_2_O_2_,and ABA treatments. As shown in Figure 4, there were wide variations in accumulation of different mRNAs following different types of stress. For salt treatment most of the transcript levels of *Di19* genes increased and remained constant during the first 12 h of treatment. *GmDi19-5* was upregulated more than 10-fold after 12 h of salt treatment (Figure 4A). Drought and H_2_O_2_ stresses also significantly modulated the expressions of most *Di19* genes. As shown in Figure 4B, transcripts of *GmDi19-5* and *GmDi19-6* were increased by more than 5-fold after 5 h of drought stress. Figure 4C showed that transcript levels of *Di19* genes, except *GmDi19-2*, instantaneously increased by more than 2-fold with H_2_O_2_ treatment; after 5 h *GmDi19-3* and *GmDi19-6* were upregulated by more than 5-fold. With ABA treatment, *Di19* transcript levels were increased more than 3-fold, except for *GmDi19-6* (Figure 4D). The transcript of *GmDi19-5* was increased more than 6-fold at 0.5 h of ABA stress.

Due to relatively high up-regulated transcript levels under stress treatments (Figure 4), *GmDi19-5* was selected for investigation. The full-length cDNA of *GmDi19-5* was comprised of 1252 bp with a 648 bp open reading frame and the deduced protein contained 215 amino acid residues with a predicted molecular mass of 24.07 kD (Table 1; Supplementary Dataset S3). Serine, threonine, and tyrosine phosphorylation sites were all found in GmDi19-5 protein (Supplementary Table S5).

### ABA and H_2_O_2_ were involved in induction of GmDi19-5 under stress treatments

**Figure 5:**
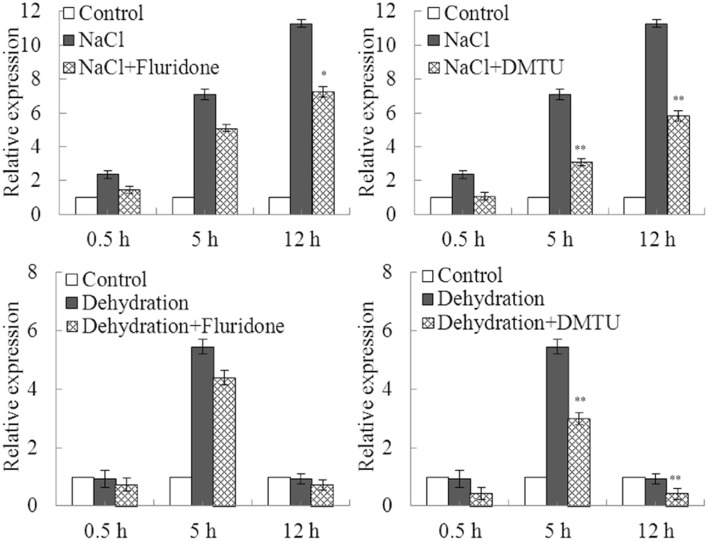
**Effects of pretreatment with inhibitor of ABA and H_2_O_2_ on the expression of *GmDi19-5* under abiotic treatments**. ABA inhibitor (Fluridone) and H_2_O_2_ inhibitor (DMTU) on expression of *GmDi19-5* in soybean seedlings exposed to dehydration and salt stresses. Mean values and standard errors (bar) were shown from three independent experiments. ^*^ and ^**^ indicated significant expression differences in comparison with expression without inhibitor pretreatment at 0.01 < *P* < 0.05 and *P* < 0.01, respectively (*t*-test).

To explore whether ABA and H_2_O_2_ were involved in up-regulation of *GmDi19-5* under drought and salt stresses, fluridone, and DMTU were chosen as ABA and H_2_O_2_ inhibitors, respectively (Hu et al., 2012; Ma et al., 2014; Yoshida et al., 2014; You et al., 2014; Zhang et al., 2014). Treatment with fluridone or DMTU had no effect on transcript of *GmDi19-5* under normal treatments (Figure 5). Pretreatment with the fluridone inhibitor partially prevented up-regulation of *GmDi19-5* under NaCl, while fluridone was not so effective in block of up-regulation of *GmDi19-5* under drought stress in soybean. Pretreatment with the DMTU inhibitor partially prevented up-regulation of *GmDi19-5* under NaCl or drought stresses in soybean. Therefore, ABA was involved in up-regulation of *GmDi19-5* under salt stresses, and H_2_O_2_ was involved in up-regulation of *GmDi19-5* under both salt and drought stresses.

### The activity of *GmDi19-5* promoter was driven by various stresses

**Figure 6:**
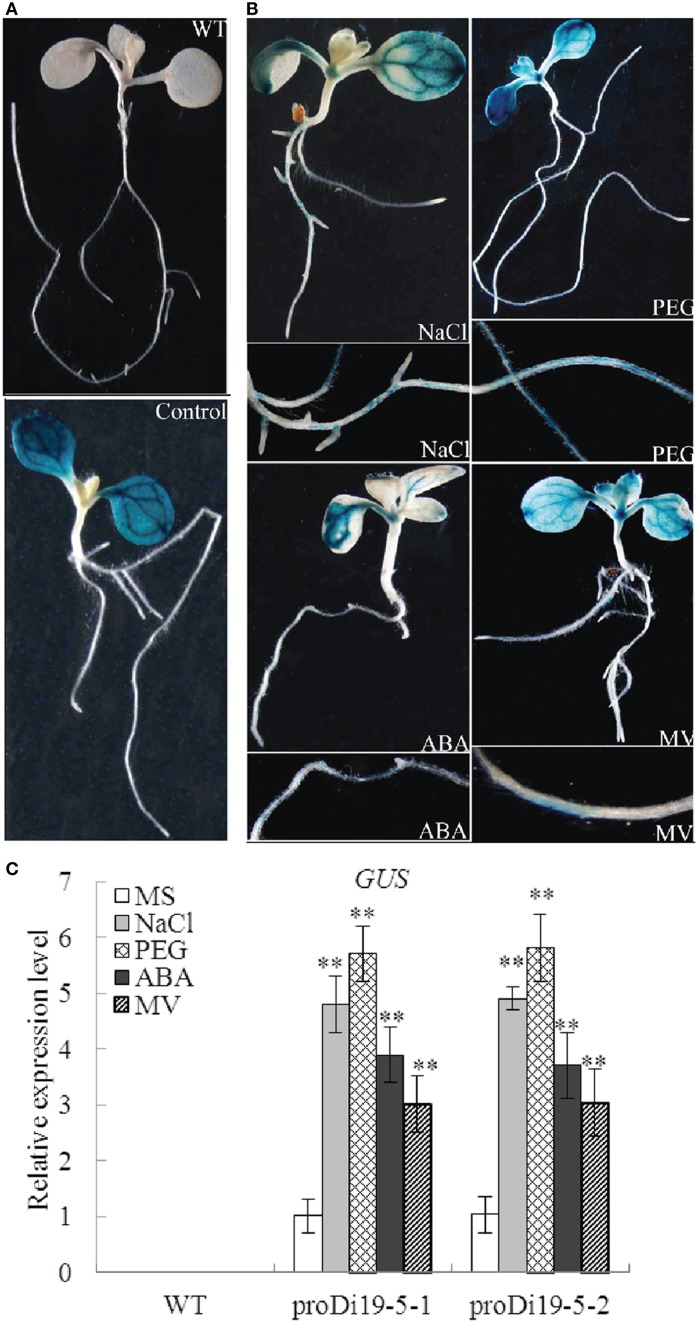
**Analysis of *GmDi19-5* promoter activities under abiotic treatments**. (A) 10-d-old seedlings of WT and transgenic plants. **(B)** Transgenic seedlings treated with NaCl, PEG, ABA, and MV. **(C)** qRT-PCR analysis of expression of *GUS* gene in proDi19-5-GUS transgenic *Arabidopsis* plants under NaCl, PEG, ABA, and MV stress treatments. ^**^ indicated significant differences in comparison with the WT lines at *P* < 0.01 (*t*-test).

To investigate the activity of *GmDi19-5* promoter, two homozygous transgenic *Arabidopsis* lines (proDi19-5-1 and proDi19-5-2) were selected for phenotypic analysis under stress treatments. Under normal growth conditions, GUS staining revealed that activity of GUS gene driven by *GmDi19-5* promoter was detected throughout cotyledons, especially in the vascular bundles of cotyledons (Figure 6A). After NaCl, PEG, ABA, and MV treatments, GUS activities significantly increased in roots, leaf primordium, and young leaves (Figure 6B). In addition, the expression level of *GUS* gene in the transgenic plants with NaCl, PEG, ABA, and MV treatments was remarkably stronger than those without NaCl, mannitol, or ABA treatment (Figure 6C).

### The *GmDi19-5* protein was localized in the nucleus

**Figure 7:**
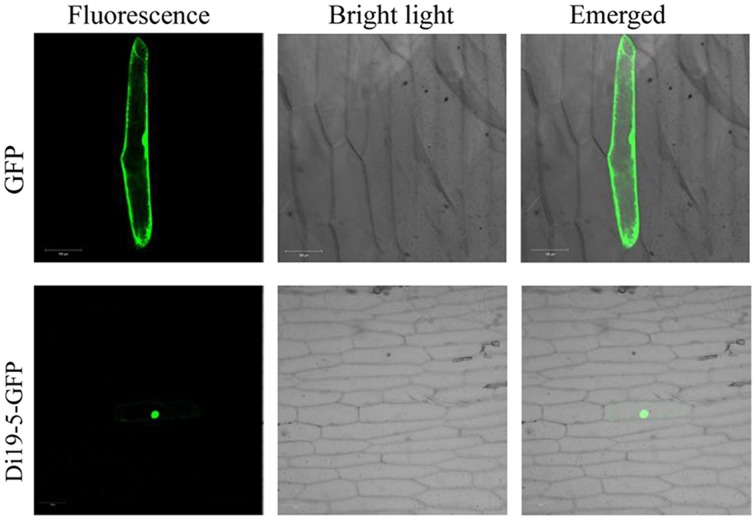
**Subcellular localization of GmDi19-5 protein**. GFP and GmDi19-5-GFP represented subcellular localization of the control 16318GFP and GmDi19-5 in onion epidermal cells, respectively.

The predicted GmDi19-5 protein contained one conserved NLS (97–118 amino acids) and one conserved NES (117–125 amino acids) (Supplementary Table S2). To determine the cellular localization of GmDi19-5 protein, the *GmDi19-5* gene was cloned into the p16318GFP vector downstream of the constitutive CaMV 35S promoter and upstream of the *GFP* gene to create the GmDi19-GFP fusion construct, which was then transformed into onion epidermal cells. Green fluorescence of GmDi19-5-GFP was mainly in the nucleus, whereas GFP in the control was uniformly distributed throughout the cell (Figure 7).

### Gmdi19-5 might be involved in the interaction with GmLEA3.1

**Figure 8:**
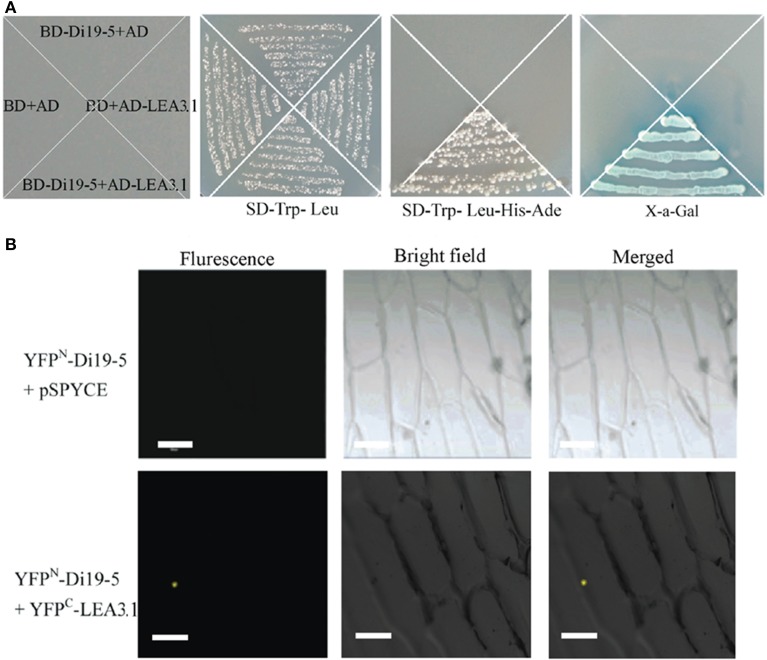
**Interaction of GmDi19-5 with GmLEA3.1. (A)** Yeast two-hybrid interaction assay. Vectors were co-introduced into yeast in different combinations: 1, pGADT7 and pGBKT7; 2, pGBKT7-GmDi19-5 and pGADT7; 3, pGBKT7 and pGADT7-GmLEA3.1; 4, pGBKT7-GmDi19-5 and pGADT7-GmLEA3.1. Transformants were placed on selection medium and grown for 4 d. **(B)** *In vivo* BiFC assay. The expression of GmDi19-5 alone (YFP^N^-GmDi19-5 + pSPYCE) was used as the control.

Interactions of proteins with other proteins are important for most biological functions. Search for interacting partners is necessary to understand the function of GmDi19-5. One positive interactor late embryogenesis abundant (LEA) protein (Glyma10g02210) was screened and identified using the yeast two-hybrid system. BLASTN and BLASTP analysis in soybean database revealed that this protein possessed the full ORF and the conserved LEA3 protein domain (Pfam 02987). Therefore, it was named as GmLEA3.1 protein. GmLEA3.1 encoded a predicted product of 95 amino acid residues with a molecular mass of 10.01 kD. In yeast two-hybrid screening, strong growth on SD-Trp-Leu-Ade-His medium and activity of the reporter gene were observed only in yeast cells co-transformed with pGBKT7-GmDi19-5 and pGADT7-GmLEA3.1 vectors (Figure 8A), indicating interaction of GmDi19-5 with GmLEA3.1 in yeast cells.

Further, we used the BiFC technology to investigate the interaction in plant cells. In contrast to the almost total absence of fluorescence in the negative control (GmDi19-5-YFP^N^ and empty vector pSPYCE), interaction of GmDi19-5-YFP^N^ (N-terminal fragment of yellow fluorescent protein) and GmLEA3.1-YFP^C^ (C-terminal fragment of yellow fluorescent protein) was observed mainly in the nucleus of onion cells (Figure 8B).

### Phenotypes of *GmDi19-5* transgenic *arabidopsis*

**Figure 9:**
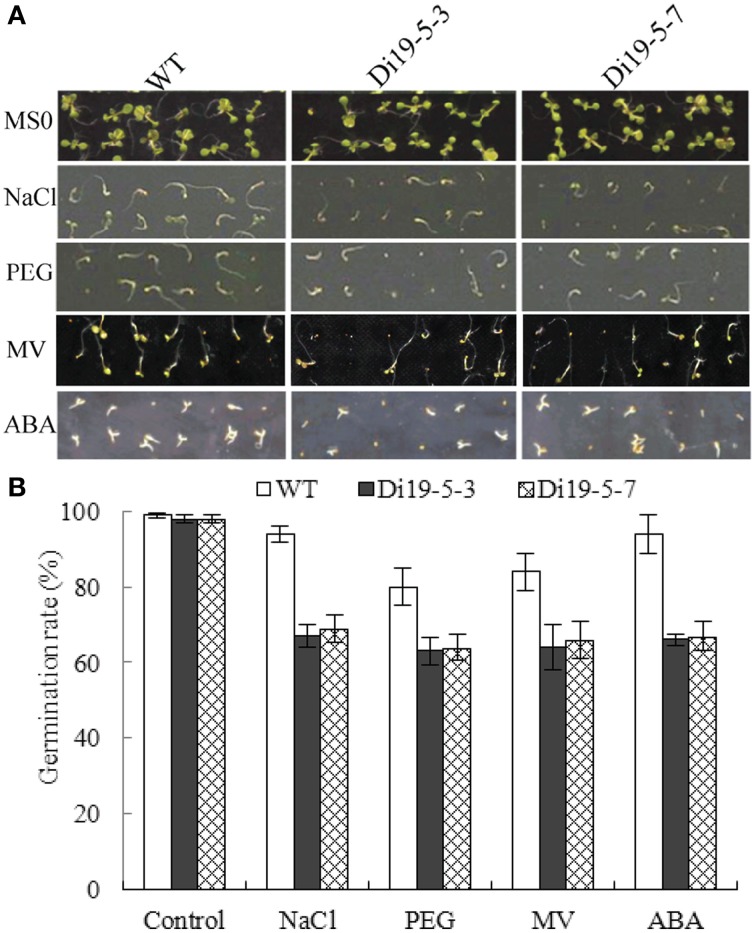
**Effect of salt, drought, oxidative and ABA stresses on seed germination of *GmDi19-5* transgenic and WT seeds. (A)** Germination rate of WT and transgenic seeds on MS medium with or without NaCl, PEG, MV, and ABA. **(B)** Statistics analysis of germination rate. Mean values and standard errors (bar) were shown from three independent experiments.

To investigate the function of *GmDi19-5*, two homozygous constitutively overexpressing *Arabidopsis* lines (Di19-5-3 and Di19-5-7) with higher *GmDi19-5* expression were selected for phenotypic analysis under NaCl, PEG, MV, and ABA stress treatments. On MS medium alone, no obvious difference was observed between the transgenic and wide type (WT) seeds. When sown on MS medium containing 50 mM NaCl, *GmDi19-5* transgenic seeds germinated much later than WT seeds. After 5 d, approximately 94% of WT seeds germinated compared to 67% for transgenic seeds. After sowing on MS medium containing 2% PEG for 5 d, approximately 80% of the WT seeds germinated, compared to about 64% for transgenic seeds. The ultimate germination rate of *GmDi19-5* transgenic seeds was slightly lower than that of WT seeds. The germination rate on MS medium containing 1.0 μM MV was also analyzed. Germination of *GmDi19-5* transgenic seeds was approximately 64% compared to 84% for WT. The germination rate on MS medium containing 1.5 μM ABA was again similar to levels observed with other stress treatment experiments. Treatment with ABA decreased the germination of *GmDi19-5* transgenic plants to approximately 66%, whereas WT retained a 94% germination rate under the same ABA concentration (Figure 9).

**Figure 10:**
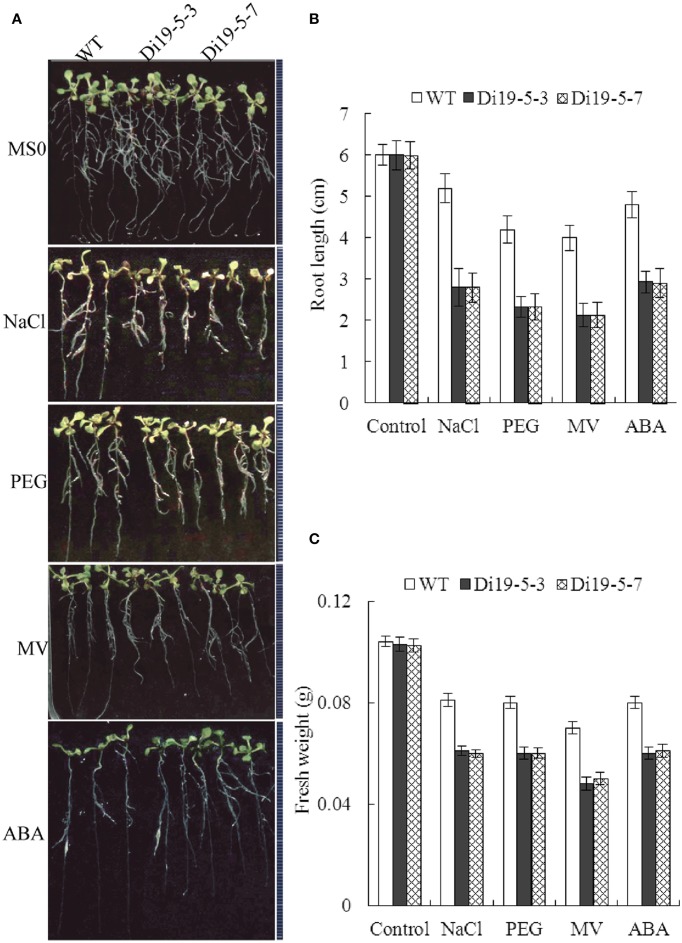
**Effect of salt, drought, oxidative, and ABA stresses on root length of *GmDi19-5* transgenic and WT seedlings. (A)** Root length of seedlings were transferred to a medium with or without NaCl, PEG, MV, and ABA before the images were taken. Statistics analysis of root length **(B)** and fresh weight **(C)**. Mean values and standard errors (bar) were shown from three independent experiments.

Root growth of *GmDi19-5* transgenic *Arabidopsis* seedlings was also investigated on MS media containing NaCl, PEG, MV, and ABA. As shown in Figure 10, when the seedlings were grown on MS medium supplemented with 100 mM NaCl or 4% PEG for 5 d, primary root growth was significantly retarded compared to WT. When the seedlings were grown on MS medium supplemented with 5 μM MV or 10 μM ABA for 5 d, the root lengths and fresh weight of the transgenic seedlings were less than those of WT. Seed germination and post-germination assays revealed increased sensitivity of the transgenic plants overexpressing *GmDi19-5* under NaCl, PEG, MV, and ABA stress conditions.

### *GmDi19-5* regulated stress-responsive gene expression

**Figure 11:**
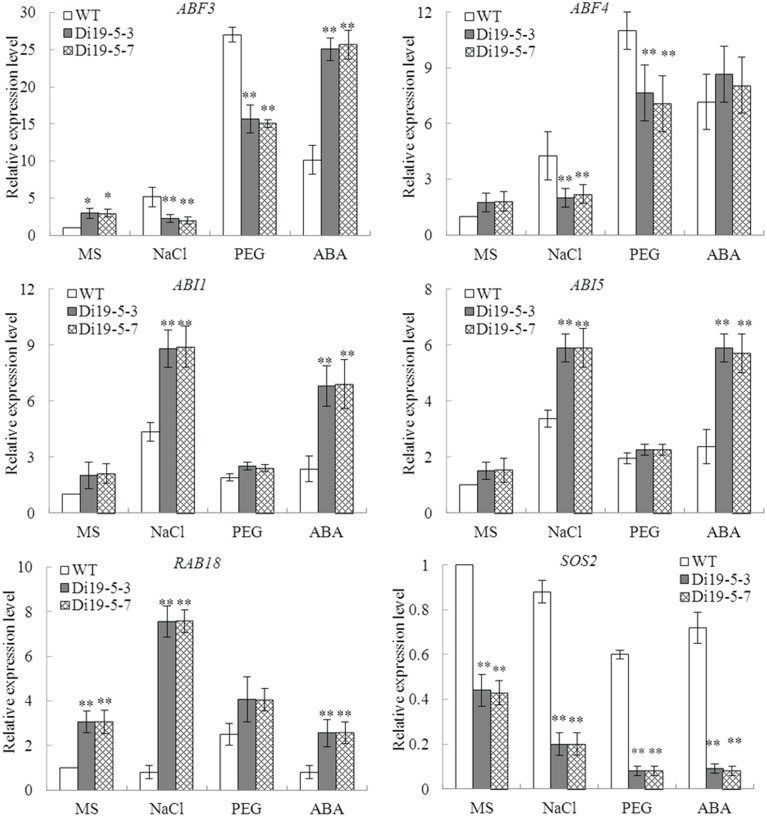
**Expression of stress-responsive genes in *GmDi19-5* transgenic *Arabidopsis***. ^*^ and ^**^ indicated significant differences in comparison with the WT lines at 0.01 < *P* < 0.05 and *P* < 0.01, respectively (*t*-test).

To elucidate the possible molecular mechanism in stress response, the expressions of six stress-response genes (*ABF3, ABF4, ABI1, ABI5, RAB18*, and *SOS2*) were investigated in constitutively overexpressing *GmDi19-5 Arabidopsis* lines (Di19-5-3 and Di19-5-7) and WT plants under normal and stress conditions. Under normal conditions, transcript levels of the *ABF3* and *RAB18* accumulated to much higher level in the transgenic lines than WT plants, while transcript level of *SOS2* in transgenic lines were remarkably lower than WT plants. Under NaCl and PEG treatments, expression levels of *ABF3, ABF4*, and *SOS2* in the transgenic lines were lower than those in WT. Under ABA stress conditions, *ABI1, ABI5*, and *RAB18* showed increased expression in the transgenic plants relative to WT plants and *SOS2* was substantially lower in transgenic lines than in WT plants (Figure 11).

## Discussion

Di19 family was involved in plant stress responses and development (Kang et al., 2005; Milla et al., 2006; Parkinson et al., 2009; Li et al., 2010a,b; Liu et al., 2013b). However, further investigation was still needed to elaborate Di19 cascades and functions. This provided an impetus for investigation of the biological roles and interacting proteins of Di19 protein family in soybean.

### Functional divergence of *Di19* genes

Di19 proteins, encoded by small multigenes, were hydrophilic, low molecular weight, and stress-responsive proteins. Domain compositions and gene structure of individual group tended to preserve similar motif compositions and relatively constant exon-intron compositions during evolution (Figures 2, 3; Supplementary Figures S1, 2). Phosphorylation sites and conserved cysteine, leu, aspartic acid, and N-myristoylation sites were found in soybean Di19 proteins, which may play important role in keeping the protein structure (Figure 1; Supplementary Table S5). Despite evolutionarily well conserved, Di19 proteins presented amino acid diversity outside the three conserved regions (Figure 1).

Di19 was involved in multiple stress responses in plants (Milla et al., 2006; Parkinson et al., 2009; Li et al., 2010a,b; Liu et al., 2013b). *Arabidopsis Di19* family genes displayed various transcriptional regulations in response to specific abiotic stress, including cold, drought, osmotic, oxidative, and salt stresses, in roots or shoots (Supplementary Figure S3; Supplementary Table S7). Promoter analysis showed there were many abiotic stress-related elements in the upstream region of soybean *Di19* family genes (Supplementary Table S6). Expression profiles analysis revealed that transcripts of soybean *Di19* genes were increased by salt, drought, oxidative, and ABA stresses (Figure 4). Meanwhile, GUS activity driven by the *GmDi19-5* promoter tended to increase under NaCl, PEG, ABA, and MV treatments (Figure 6). It was reported that several genes responsive to salt and drought might be involved in ABA and H_2_O_2_ signaling pathways that were conserved components of these stress signal pathways (Hu et al., 2012; Ma et al., 2014; Yoshida et al., 2014; You et al., 2014; Zhang et al., 2014). Pretreatment with the inhibitors of these pathways prevented up-regulation of *GmDi19-5* in NaCl- and drought-treated soybean seedlings (Figure 5). Thus, it is plausible that *GmDi19-5* could be part of the ABA- and ROS-mediation process. However, more research is needed to confirm this mechanism through other biological technologies.

It was demonstrated that salt and drought stresses were able to induce ABA biosynthesis and trigger ABA-dependent signaling pathways, and ABA could maintain seed dormancy, prevented germination and inhibited seedling growth (Finkelstein et al., 1998, 2002; Leung and Giraudat, 1998; Achard et al., 2006; Piskurewicz et al., 2008; Bari and Jones, 2009). In cotton, Di19-like genes *GhDi19-1* and *GhDi19-2* were identified to be positive nuclear regulators in the ABA signaling pathway (Li et al., 2010a). Wheat *TaDi19A* was positively regulated by ABA signaling and was responsive to NaCl and high osmotic stress (Li et al., 2010b). *GmDi19-5* affected the expression of genes related to ABA/stress signaling in transgenic plants, including *ABF3, ABF4, ABI1, ABI5, RAB18*, and *SOS2* genes (Figure 11). *ABF3* and *ABF4* were two bZIP transcription factors which positively regulated ABA signaling. The constitutive overexpression of *ABF3* or *ABF4* in *Arabidopsis* exhibited enhanced drought tolerance and ABA hypersensitivity (Kang et al., 2002). The relative expression levels of these two genes were higher after ABA treatment. *ABI1* encoded one ser/thr protein phosphatase of the PP2C family that acted as negative regulator of ABA response (Koornneef et al., 1989). *ABI5* was one bZIP transcription factor, positively regulating ABA-mediated control of seed germination and early seedling development (Leung and Giraudat, 1998). The level of *ABI1* and *ABI5* were significantly increased under ABA treatment. The transcription level of known ABA up-regulated gene *RAB18* was also elevated in *GmDi19-5* transgenic plants. These findings suggested that ABA was more effectively synthesized in transgenic plants than in WT plants. *GmDi19-5* might act a positive regulator in ABA response. In addition, *ABF3, ABF4*, and *SOS2* were significantly low in *GmDi19-5* transgenic plants after NaCl and PEG treatments. Overexpression of *GmDi19-5* displayed increased sensitivities to salt, drought, and oxidative stress during the germination and seedling stages in *Arabidopsis* (Figures 7, 8). Recently, it was reported that *AtDi19-3* displayed drought- and salt-sensitive phenotype in overexpression lines (Qin et al., 2014). Similar to *AtDi19-3, GmDi19-5* might act as a negative regulator in response to salt and drought stresses (Qin et al., 2014).

### Di19s might be involved in the interactions with various proteins

Zinc-finger domain is involved in protein-DNA interactions, protein-protein interactions between cytoskeleton dystrophin and calmodulin, and between transcriptional adapters (such as CREB-binding proteins and p300) and transcriptional activators (Dure, 1993; Davies et al., 1996; Takatsuji, 1999; Searles et al., 2000; Wolfe et al., 2000). The Cys2/His2 zinc finger-like domain may have a similar function in protein-protein interaction. LEA proteins possessed high hydrophilicity and chaperone-like activity and involved in stress resistance in plants, such as chilling, drought, and high salinity (Battaglia et al., 2008; Bies-Etheve et al., 2008; Battaglia and Covarrubias, 2013). Physical interaction with other proteins was one of the important features of LEAs, such as between hydrophilic proteins and LEA proteins (Hanin et al., 2011; Cuevas-Velazquez et al., 2014). Maize LEA protein Rab17 interacted with and was phosphorylated protein kinase CK2 (Riera et al., 2004). *Opuntia streptacantha* LEA proteins were shown to form dimer (Hernández-Sánchez et al., 2014). LEAs belonged to a multigene family, which was classified into seven groups based on expression patterns and sequences. Recently, there were extensive correlative data linking the expression of Group 3 LEA (LEA3) proteins with tolerance suggested various biochemical mechanisms for LEA3s, such as the repair of improperly folded proteins as a chaperone, the binding of metal ions, the stabilization of membrane structure and the increase of cellular mechanical strength through the generation of filaments (Browne et al., 2002; Tolleter et al., 2007; Tunnacliffe and Wise, 2007). We found that GmDi19-5 interacted with GmLEA3.1 (Figure 8), thus we might suppose that GmLEA3.1 protein might improve the stability of GmDi19-5 protein.

In addition, GmDi19-5 contained a conserved NLS in which putative kinase phosphorylation sites (Serine and Tyrosine) were located (Supplementary Table S5). *Arabidopsis* AtDi19-1 interacted with and was phosphorylated by AtCPK11 at the NLS-containing motif (Milla et al., 2006). AtDi19-4 was phosphorylated by AtCPK3 at the NLS-containing motif with Serine 116 being identified as one of the putative phosphorylated sites (Milla et al., 2006). It was possible that GmDi19-5 protein might interact with other proteins or be modulated at the post-translational level by changing their phosphorylation states to enhance the abiotic stress in plants. Interactions with different factors may be essential for activation of Di19 proteins or one mechanism distinguishing the different functions of the Di19 members. Therefore, their function and interacting proteins still need to be studied in detail, which will help to elaborate Di19 cascades.

## Author contributions

ZSX coordinated the project, conceived and designed experiments, and edited the manuscript. ZJF conducted the bioinformatic work, generated and analyzed data, and wrote the first draft. XYC and XYC performed experiments and analyzed the data. MC and GXY managed reagents and provided analytical tools. YZM and GYH contributed with valuable discussions. All authors have read and approved the final manuscript.

### Conflict of interest statement

The authors declare that the research was conducted in the absence of any commercial or financial relationships that could be construed as a potential conflict of interest.

## Supplementary material

Dataset 1 **Fasta file of soybean Di19 protein and conserved domain sequences**.

Dataset 2 **Fasta file of all *Arabidopsis* and rice Di19 protein conserved regions**.

Dataset 3 **Fasta files of soybean *Di19-5* gene and protein sequences**.

Supplementary Table S1 **List of primers used in this paper**.

Supplementary Table S2 **NLS and NES sequences identified in soybean Di19 family proteins by the PSORT and NetNES softwares**.

Supplementary Table S3 **Nomenclature for Di19s in soybean, *Arabidopsis*, rice, maize, and *Brachypodium***.

Supplementary Table S4 **Conserved motifs identified for the Di19 proteins from soybean, *Arabidopsis*, rice, maize, and *Brachypodium* by MEME software**.

Supplementary Table S5 **Phosphorylation sites predicted in soybean Di19 family proteins by NetPhos 2.0 Server softwares**.

Supplementary Table S6 **Prediction *cis*-elements of soybean Di19 family genes**.

Supplementary Table S7 **Expression profiles of *Arabidopsis* Di19 genes in response to various stress treatments**. The data was downloaded from http://bar.utoronto.ca/efp/cgi-bin/efpWeb.cgi.

Supplementary Figure S1 **Variation in motif clades of Di19 proteins from soybean, *Arabidopsis*, rice, maize, and *Brachypodium***. The MEME motifs were shown as different-colored boxes.

Supplementary Figure S2 **Logos of the conserved Di19 domain alignments of *Arabidopsis*, rice, maize, and *Brachypodium***.

Supplementary Figure S3 **Expression profiles of *Arabidopsis Di19* genes in response to various stress treatments**. Hierarchical clustering of differential gene expression in response to cold **(A)**, drought **(B)**, heat **(C)**, osmotic **(D)**, oxidative **(E)**, and salinity **(F)** stresses.

## Abbreviations

ABA: abscisic acid
Di19: drought-induced
DMTU: dimethyl thiourea
GFP: green fluorescent protein
MV: methylviologen
qRT-PCR: quantitative real-time PCR
WT: wild-type.
