# Supplementary figures and images for: The soybean GmDi19-5 interacts with GmLEA3.1 and increases sensitivity of transgenic plants to abiotic stresses
Source: Front Plant Sci. 2015 Mar 24;6:179. doi: 10.3389/fpls.2015.00179 (PMC4371698; doi:10.3389/fpls.2015.00179)

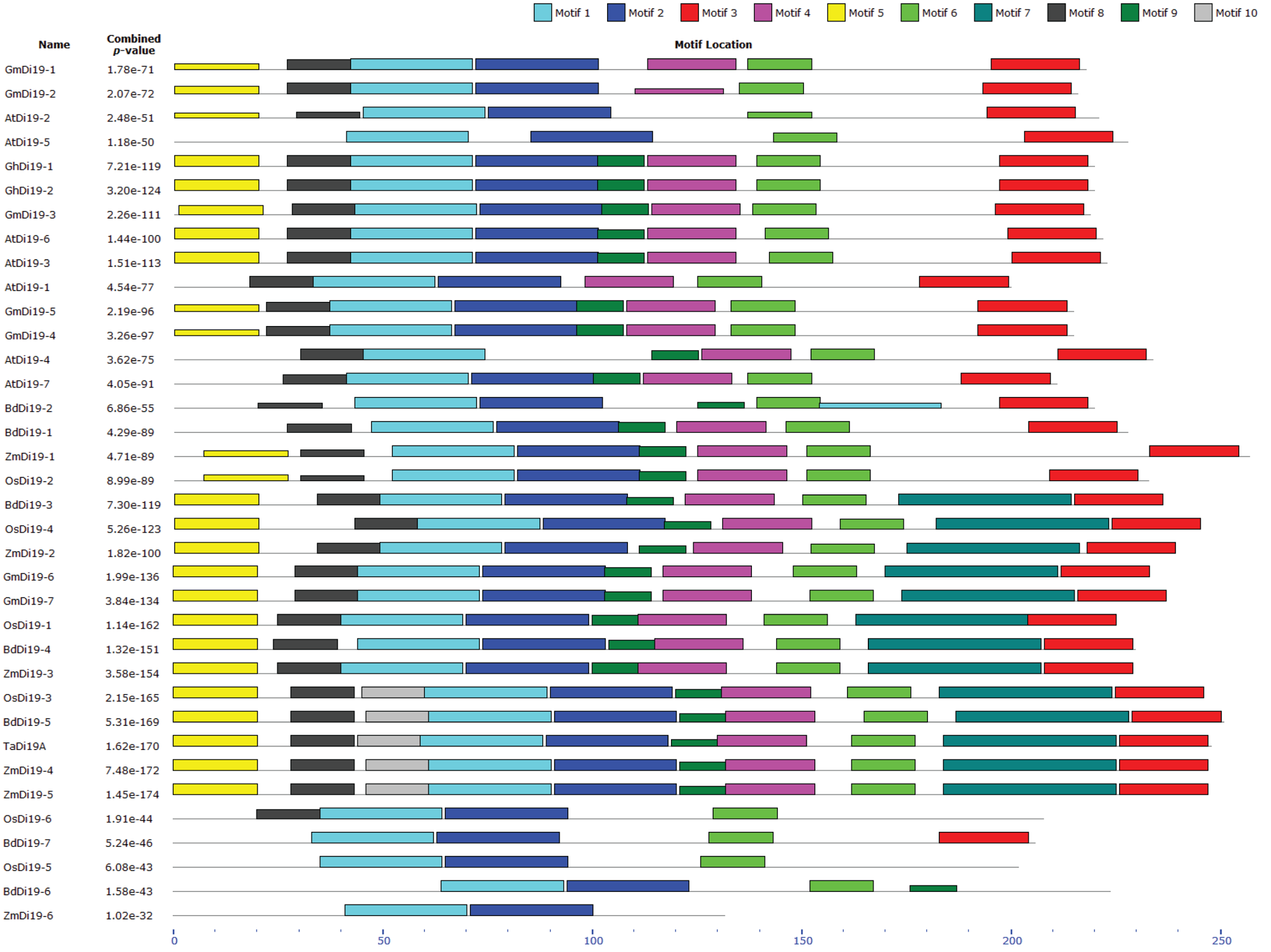

Supplement: Dataset 1 — Fasta file of soybean Di19 protein and conserved domain sequences. [file DataSheet1.ZIP › Image 1.TIF]

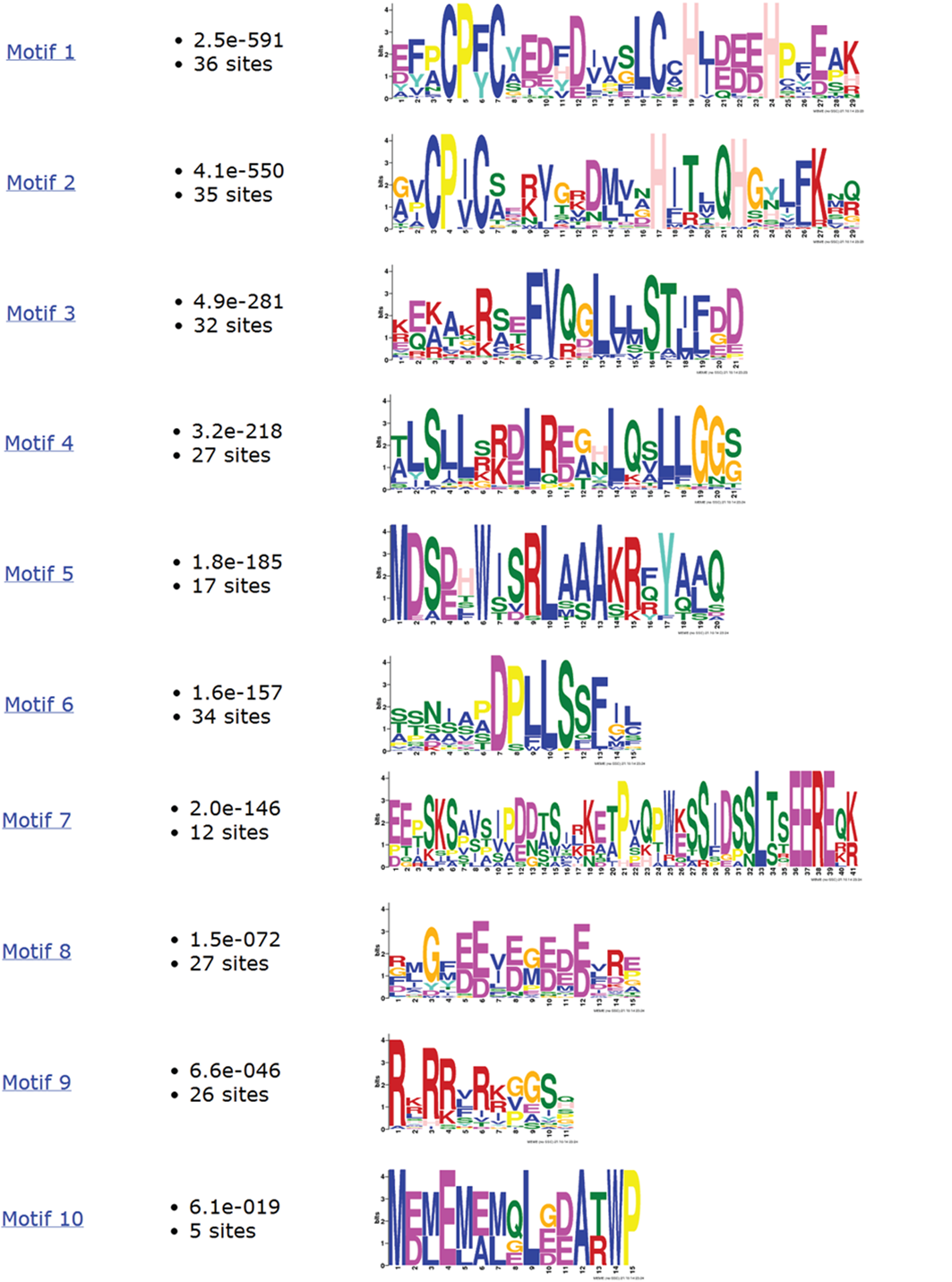

Supplement: Dataset 1 — Fasta file of soybean Di19 protein and conserved domain sequences. [file DataSheet1.ZIP › Image 2.TIF]

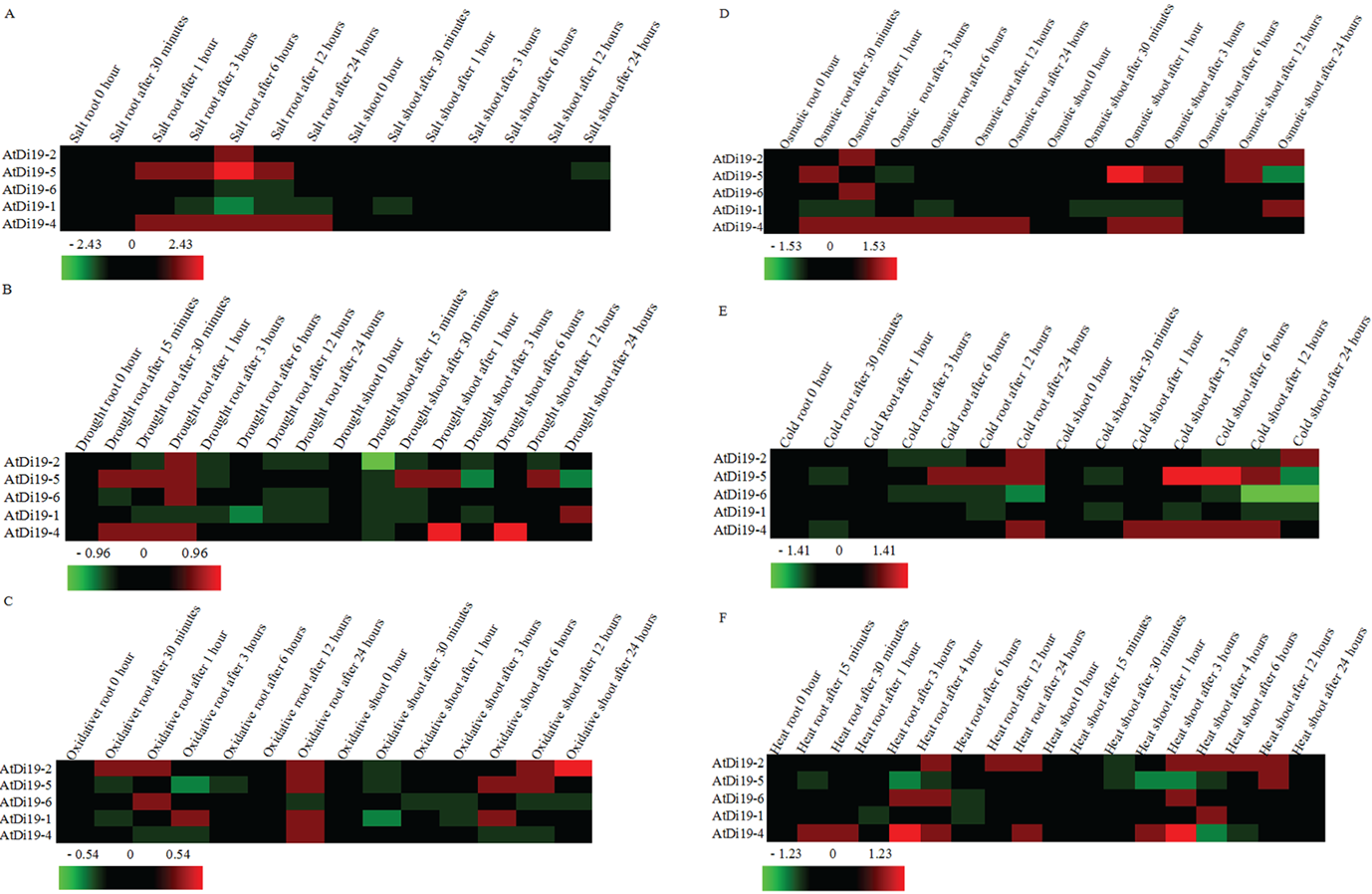

Supplement: Dataset 1 — Fasta file of soybean Di19 protein and conserved domain sequences. [file DataSheet1.ZIP › Image 3.TIF]
