# Supplementary material for: The soybean GmDi19-5 interacts with GmLEA3.1 and increases sensitivity of transgenic plants to abiotic stresses
Source: Front Plant Sci. 2015 Mar 24;6:179. doi: 10.3389/fpls.2015.00179 (PMC4371698; doi:10.3389/fpls.2015.00179)
Supplement: Dataset 1 — Fasta file of soybean Di19 protein and conserved domain sequences. [file DataSheet1.ZIP › Data Sheet 1.DOC]

GmDi19 - predicted full length protein sequences (note that in some cases more than one gene model may exist).

>GmDi19-1 Glyma03g37530

MEDETLSFVLSTPSRSYQSRLKSHFELFIDLDEVNGDEELRTAYPCPFCAENFDLLELCCHVDLDHPIEAKSGICPVCTLWIGTNMVHHIAAQHGNLLKSHLKSKCYKDEPYPALSFSSKGERDGHWQSFSTGLSPTTSKAASDPWLSFLCGPSAVDECENVQPDSSSEVSIEEIHSNDNVLERDVQPSLSDKDQIEKAQHSKFVQGLLMSTILDPDF*

>GmDi19-2 Glyma19g40150

MEDETLSLGLSTASRSYQSRLKSHFELFIDFDEVNGDEELRTAYPCPFCTEDFDLLELCCHIDLDHPVEAKSGICPVCTMWIGTNMVDHIAAQHGNLFKSQLKSKCYKDESYPALSFSRKGEHWQSFSTGLSAMSTSKAASDPWLSFLCGASAVDERKNVQPDSSREVSIEELHSNDKVLVRDVQPSLSDKDQIEKAQRSKFVQGLLMSTILDPDF*

>GmDi19-3 Glyma10g29030

MDGDSSWSARLSSASRRYQSALQSRSDMFMGFDENDGDDDIREEFLCPFCSEYFDIVGLCCHIDEEHPMEAKNGVCPVCALRVGVDMVAHITLQHGSIFKMQRKRKSRKGGSYSTLSLLRKELREGNLQSLFGGSSCIVSSSNADPLLSSFILPLANEHASSQPHLHTETRSSKKCLDETVSTRNVETSTLSVKDKEEKEKRCEFVQGLLLSTILDDNL*

>GmDi19-4 Glyma15g15560

MDSDSWISTRLSSSSRRHHYRSDLYAEESEGNDDFRAEFLCPFCAEDYDVVSLCCHIDDHHPIQAKNGVCPICGKKVGVDLVGHFTTQHGNFLRVQRKRRVRKGGSASTISILRKELQEGALQSLLGGSSYLASSNSEPDPLLSSFMFNPVVADESVSATPPSTEDALVKESSKDDFLKRKPQQLQLSEEDQVEKARRFEFVQGLLMSTILDDKL*

>GmDi19-5 Glyma09g04490

MDSDSWISTRMSSSSRRYHSRSDLYVEESEGSDDFRAEFLCPFCAEDYDVVSLCCHIDDHHPIQAKNGVCPICGKKVGLDLVGHITTQHGNFLRVQRKRRVRKVGSGSTMSILRKELREGALHSLLGGSSYLASCNSEPDPLLSSFMFNPAVTDDSASAKPPSVEDALVKESSKDDFLERKPQQLQLSEEDQVEKARRFEFVQGLLMSTILDDML*

>GmDi19-6 Glyma07g32120

MDSDFWTSRLAAAKRQYTLQHHHPNSHLDRLGIDDFDVEEEVRPDFPCPYCYEDFDIASLSSHLEDEHSCESRVTICPICSVKVARDMLNHITLQHGHLFKLQRRRRLRRVAIPNSQTLSLLGRDLREAHLQVLLGGGGGYRSNSAAVSNAAATDPFLSSFILNFPACEAEEISKSVVTSADDSSSKNATPVHIWKSSFDPSLSTEEREKRMRQAAGRSGFVQDLFLSTLLGE*

>GmDi19-7 Glyma13g24420

MDSDFWTSRLAAAKRQYTLQHHHPNSHLDRLGIDDFDMEEEVRPDFPCPYCYEDFDIASLCSHLEDEHSCESRVTICPICSDKVARNMLSHITLQHGHLLKLQRRRRLRRVAIPNSQTLSLLGRDLREAHLQVLLGGGGGGGYRSNSVAAAVSNAAATDPFLSSFILNFPACEAEEISKSVVTSAENSSAKNATPEHIWKSSFGPSLSTEEREKRMRQAAGRSGFVQELFLSTLLGE*

GmDi19 - predicted conserved regions only.

>GmDi19-1Glyma03g37530

CPFCAENFDLLELCCHVDLDHPIEAKSGICPVCTLWIGTNMVHHIAAQ

>GmDi19-2 Glyma19g40150

CPFCTEDFDLLELCCHIDLDHPVEAKSGICPVCTMWIGTNMVDHIAAQ

>GmDi19-3Glyma10g29030

CPFCSEYFDIVGLCCHIDEEHPMEAKNGVCPVCALRVGVDMVAHITLQ

>GmDi19-4Glyma15g15560

CPFCAEDYDVVSLCCHIDDHHPIQAKNGVCPICGKKVGVDLVGHFTTQ

>GmDi19-5Glyma09g04490

CPFCAEDYDVVSLCCHIDDHHPIQAKNGVCPICGKKVGLDLVGHITTQ

>GmDi19-6Glyma07g32120

CPYCYEDFDIASLSSHLEDEHSCESRVTICPICSVKVARDMLNHITLQ

>GmDi19-7Glyma13g24420

CPYCYEDFDIASLCSHLEDEHSCESRVTICPICSDKVARNMLSHITLQ
