# Supplementary material for: The soybean GmDi19-5 interacts with GmLEA3.1 and increases sensitivity of transgenic plants to abiotic stresses
Source: Front Plant Sci. 2015 Mar 24;6:179. doi: 10.3389/fpls.2015.00179 (PMC4371698; doi:10.3389/fpls.2015.00179)
Supplement: Dataset 1 — Fasta file of soybean Di19 protein and conserved domain sequences. [file DataSheet1.ZIP › Data Sheet 2.DOC]

These are the Arabidopsis Di19, conserved regions only.

AtDi19-1 AT1G56280.1

CPFCAESYDIIGLCCHIDDEHTLESKNAVCPVCSLKVGVDIVAHITLQHGSLFKLK

>AtDi19-2 AT1G02750.1

CPFCASDYDLVELCHHIDEEHRHEANNGICPVCSKRVKMHMVDHITSH

>AtDi19-3 AT3G05700.1

CPFCSDYFDIVSLCCHIDEDHPMEAKNGVCPVCAVRVGVDMVAHITLQH

>AtDi19-4 AT3G06760.1

CPFCAEDFDIVGLCCHIDEEHPVEAKNGVCPVCTKRVGLDIVGHITTQ

>AtDi19-5 AT4G02200.1

CPFCSDDYDLVELCHHIDEEHQLDANNGICPVCSRRVKMHMVDHITTQ

>AtDi19-6 AT5G26990.1

CPFCSDYFDIVSLCCHIDEDHPMDAKNGVCPICAVKVSSDMIAHITLQ

>AtDi19-7 AT5G49230.1

CPFCADEFDIVGLCCHIDVNHPVEAKNGVCPVCTKKVGLDIVGHITTQ

These are the rice Di19, conserved regions only.

>OsDi19-1 Os05g0562200

CPYCYEDHDVVSLCAHLEEEHPFEPHAAPCPICSDKIAKDMLNHITVQH

>OsDi19-2 Os05g0358000

CPFCGEDFDFVAFCCHVDDEHAVEAKSGVCPICATRVGVDLIGHLTMQH

>OsDi19-3 Os01g0672400

CPYCYEDHDIASLCAHLEEDHPYEPHTSPCPICFEKITRDMLNHITMQH

>OsDi19-4 Os02g0304900

CPYCYEDHDITSLCAHLEDEHPFESKVVACPVCSARISKDLLDHITLQH

>OsDi19-5 Os01g0971100

CPFCYIEVEVPFICNHLQEEHCFDTRNAVCPLCADNIGRDMGAHFRVQH

>OsDi19-6 Os05g0107900

CPFCYIEVEVHFLCDHLQEEHCFDMKNAVCPICADNLDKDTDEHFRVQH
