# Supplementary material for: The soybean GmDi19-5 interacts with GmLEA3.1 and increases sensitivity of transgenic plants to abiotic stresses
Source: Front Plant Sci. 2015 Mar 24;6:179. doi: 10.3389/fpls.2015.00179 (PMC4371698; doi:10.3389/fpls.2015.00179)
Supplement: Dataset 1 — Fasta file of soybean Di19 protein and conserved domain sequences. [file DataSheet1.ZIP › Data Sheet 3.DOC]

Fasta files of soybean Di19-5 gene and protein sequences.

>GmDi19-5 CDS

atggattccgattcttggatttccacgcgcatgtcgtcctcttcacgccgctatcattcgcgatctgatctgtacgtggaggagagtgaagggag

cgatgatttcagggcggagtttctgtgccctttctgcgccgaggattacgacgtcgtttccctctgttgccacattgacgaccaccaccctatccaagccaaaaacggggtgtgtcccatttgtgggaagaaggtgggattggatctcgttggacatattactacgcaacatgggaacttcttgagggtgcagcgtaaaaggagggtcaggaaagtaggctctggttcaacaatgtctatattgagaaaagagttacgagaaggagccttgcactcccttcttggaggttcttcatacttagcttcctgcaattctgagcctgatcctctgttatcatcttttatgtttaacccagctgtaactgatgattctgcgagtgcaaaacctccttctgttgaagatgctctagtaaaagaaagttcaaaagacgatttcttggaaagaaaacctcaacagttacagctgtcagaagaagatcaagtagagaaggctcggaggtttgaattcgtgcaaggacttctgatgtccaccattcttgatgatatgttatga

>GmDi19-5 protein

MDSDSWISTRMSSSSRRYHSRSDLYVEESEGSDDFRAEFLCPFCAEDYDVVSLCCHIDDHHPIQAKNGVCPICGKKVGLDLVGHITTQHGNFLRVQRKRRVRKVGSGSTMSILRKELREGALHSLLGGSSYLASCNSEPDPLLSSFMFNPAVTDDSASAKPPSVEDALVKESSKDDFLERKPQQLQLSEEDQVEKARRFEFVQGLLMSTILDDML*
